# More than a Feeling: Self-Esteem as a Predictor of Life Satisfaction in Adolescents: A Cross-Sectional Analysis in Saudi Arabia

**DOI:** 10.3390/children12081046

**Published:** 2025-08-09

**Authors:** Hawa Alabdulaziz

**Affiliations:** Faculty of Nursing, King Abdulaziz University, Jeddah 21589, Saudi Arabia; halabdulaziz@kau.edu.sa

**Keywords:** self-esteem, life satisfaction, adolescents, well-being Saudi Arabia

## Abstract

**Highlights:**

**What are the main findings?**

- Self-esteem was found to be a significant predictor of life satisfaction among Saudi adolescents.
- Adolescents dissatisfied with life were over seven times more likely to report low self-esteem.

**What is the implication of the main finding?**

- The study highlights the need for culturally tailored mental health interventions that focus on strengthening self-esteem to support adolescent well-being.
- The study emphasizes the value of integrated, school-based programs addressing both psychological and socio-environmental factors in adolescent development.

**Abstract:**

**Introduction:** Prior research underscores self-esteem as a core determinant of life satisfaction and overall well-being. However, few studies have examined this relationship within the unique sociocultural context of Saudi Arabia. This study assesses the relationship between self-esteem and life satisfaction in adolescents, as well as the potential moderating effects of demographic factors. **Methods:** A cross-sectional study was conducted involving 502 adolescents aged 13–18, selected from urban and rural regions of Saudi Arabia. Participants completed the Rosenberg Self-Esteem Scale and the Satisfaction with Life Scale. Data were collected via online survey platforms. Descriptive statistics, correlation analyses, chi-square tests, and multivariate logistic regressions were performed using SPSS version 26. Ethical approval was obtained, and informed consent was secured from all participants. **Results:** A significant positive correlation was found between life satisfaction and self-esteem scores (r = 0.37, *p* < 0.001). Adolescents reporting dissatisfaction with life had over seven times greater odds of low self-esteem (OR = 7.2; 95% CI: 3.75–13.83). Higher life satisfaction was associated with being in secondary education, having a family income of 10,000 Saudi Riyal (SR) or more, and living with both parents. Additionally, prior contact with a psychologist was linked to lower self-esteem and reduced life satisfaction. **Conclusions:** Findings confirm self-esteem as a strong predictor of life satisfaction among Saudi adolescents. Socioeconomic status, family structure, and previous psychological consultation also influenced outcomes. These results emphasize the critical need for culturally sensitive mental health interventions tailored to the Saudi context. Furthermore, they highlight the importance of implementing early mental health screening and support programs within schools to provide accessible and preventive care for youth.

## 1. Introduction

Adolescence constitutes a critical development period characterized by rapid biological, emotional, and psychosocial transformations [1]. During this stage, adolescents experience considerable development, develop independence and autonomy, and adapt to diverse social environments, which together contribute meaningfully to influencing mental health outcomes [1]. Recent research has highlighted the importance of self-esteem and life satisfaction in adolescent psychological adjustment [2,3]. Among the many constructs contributing to adolescent mental health, self-esteem [4,5] and life satisfaction [2,6] emerge as fundamental determinants of positive adjustment and resilience.

According to Alquwez et al. (2021) and Bahar et al. (2024), self-esteem positively influences life satisfaction and overall well-being, highlighting its developmental importance [4,5]. Self-esteem, broadly defined as an individual’s evaluative appraisal of their own worth, plays an essential role in shaping emotions, motivation, and interpersonal functioning. High self-esteem has been consistently linked with better emotional regulation, greater academic motivation, stronger social skills, and overall psychological well-being during adolescence—a period when identity development is salient [4,5]. Conversely, low self-esteem may predispose adolescents to vulnerabilities such as anxiety, depression, and maladaptive behaviors [6,7]. Other literature has established self-esteem as a foundational component of psychological resilience, particularly during adolescence [2,6]. Adolescents with greater self-esteem tend to demonstrate stronger emotional regulation, academic motivation, interpersonal competence, and overall well-being.

Moreover, life satisfaction, a key component of subjective well-being, is conceptualized as a stable, trait-like judgment of one’s overall contentment with life [7]. Life satisfaction is a key facet of subjective well-being that reflects a global, stable judgment of one’s contentment with life circumstances. Research indicates that judgments of life satisfaction are based on available information, such as emotional experiences and memories, contributing to a person’s stability over time [8,9,10]. These measures are increasingly being used as indicators in national accounts of well-being [5,11,12].

Worldwide, recent studies have consistently demonstrated a direct link between self-esteem and life satisfaction across various populations. In one study among Polish adolescents, self-esteem showed a positive connection with life satisfaction, mediated by peer communication [13]. Norwegian adolescents exhibited a strong positive association between self-esteem and life satisfaction, while loneliness was related to lower levels of life satisfaction [14]. Among Spanish adolescents, self-esteem played a mediating role between self-efficacy and life satisfaction [2]. A study in Lebanon reported that self-critical perfectionism mediated the connection between self-esteem and life satisfaction [15]. Thus, the literature underscores the significance of self-esteem in promoting life satisfaction among various age groups and cultures, suggesting potential interventions to enhance well-being.

Saudi Arabia presents a unique developmental landscape, one that balances the acceleration of modernization with time-honored cultural traditions [16,17]. Over the last decade, the country has pursued reforms in education, technology, and public life, producing rapid institutional and social change. In addition to these advances, evolving gender norms, global media exposure, changing household patterns, and heightened academic pressures have reconfigured daily experiences for adolescents [18,19]. Recent research has highlighted growing concerns about emotional distress, anxiety, and identity-related conflicts among Saudi youth, driven in part by the tensions often traced to the clash between longstanding cultural norms and rapid modernization [20,21].

However, despite these challenges, baseline information on positive psychological constructs such as self-esteem and life satisfaction remains scarce in the country. Most available studies have focused on risk factors such as internet addiction, depression, or academic burnout [18,19,20,21], but little attention has been paid to the protective psychological resources that enhance resilience and well-being. Hence, understanding these protective factors is particularly urgent in light of Saudi Arabia’s Vision 2030, which highlights youth development, quality of life, and mental health promotion as national priorities. Not only can such studies inform culturally tailored mental health programs, but they can also offer insight into how traditional values and modern pressures jointly shape adolescent development in the Gulf region.

This study contributes to the global discourse on adolescent well-being by providing evidence from a non-Western, under-researched population. The findings contribute to theory by testing the applicability of established psychological models within a culturally distinct context and provide actionable insights for policymakers, educators, and mental health practitioners focused on promoting adolescent well-being in Saudi Arabia.

Therefore, the aim of this study is to assess the relationship between self-esteem and life satisfaction in adolescents, as well as the potential moderating effects of demographic factors. Specifically, the study (1) assesses the levels of self-esteem and life satisfaction in a representative adolescent sample; (2) determines whether self-esteem is a significant predictor of life satisfaction; and (3) evaluates whether this relationship is moderated by key demographic variables.

## 2. Materials and Methods

### 2.1. Design

A cross-sectional research design was employed in this study to examine the association between self-esteem and life satisfaction among Saudi adolescents. The cross-sectional approach allows for assessment of these psychological variables and their correlations at a single point in time.

### 2.2. Participants and Sampling

A total of 590 adolescents aged 13 to 18 years from various cities in Saudi Arabia were invited to participate in the study. Of these, 502 completed the survey (response rate = 85.1%). The sample size exceeds the calculated minimum required size of 385 participants (determined using Raosoft Online Calculator software (http://www.raosoft.com/samplesize.html (accessed on 5 August 2025)) with a 5% margin of error, 95% confidence level, 20,000 population size, and 50% response distribution). This high response rate supports the representativeness of the sample and strengthens the validity and generalizability of the study findings.

A convenience sampling method was used, with recruitment conducted primarily through popular social media platforms among Saudi teenagers, including WhatsApp, Telegram, and X. These platforms were selected due to their wide reach and accessibility within the target demographic. To enhance socioeconomic and geographic diversity, recruitment efforts targeted both urban and rural populations across Saudi Arabia.

Parental involvement and consent were secured via a dual recruitment pathway to ensure ethical compliance. In some instances, study invitations were disseminated through groups or contacts with active parent or guardian participation (e.g., parent community chats), allowing for direct communication to obtain parental consent. In other instances, adolescents accessed the survey link individually and were explicitly instructed, both in the invitation and at the survey start, to discuss participation with their parent or guardian, who provided electronic informed consent prior to the adolescent’s survey completion.

This dual pathway ensured that parental or guardian consent was obtained for all participants under 18 prior to the collection of any survey data, thereby fulfilling ethical requirements for research involving minors in an online and social media context.

The inclusion criteria required participants to be residents of Saudi Arabia within the specified age range. Adolescents diagnosed with significant mental health disorders (e.g., schizophrenia, bipolar disorder) or known cognitive impairments were excluded. These exclusion criteria ensured a sample representative of generally healthy adolescents and helped avoid confounding factors and repeat participation.

During the exclusion screening procedure, participants—and their parents or caregivers in the case of minors—were asked during the initial electronic screening and consent process whether they had been diagnosed with any significant mental health or cognitive conditions. Additionally, participant identifiers (e.g., contact details) were cross-checked against a database from a previous related study conducted within six months to prevent duplicate participation and maintain sample independence.

### 2.3. Measures

Demographic information was collected to characterize the participant sample, including age, gender, educational levels of the child and parents, employment status of parents, monthly family income, living arrangements, place of residence, and history of psychological consultation.

The Rosenberg Self-Esteem Scale (RSES) is a questionnaire that assesses participants’ sense of self-worth. It consists of 10 statements related to self-esteem from aspects such as self-acceptance and self-confidence [22,23]. Five of the statements are positively worded, and the remaining five are negatively worded. Participants are required to rate each statement on a scale from “Strongly Disagree” to “Strongly Agree.” The questionnaire assigns points to each response. A higher total score implies higher self-esteem. The scale ranges from 0 to 30. Scores between 15 and 25 are within the normal range; scores below 15 suggest low self-esteem. The RSES demonstrates strong test-retest reliability (α ranging from 0.82 to 0.88 over a 2- to 4-week interval) [23]. It has been validated across diverse cultural contexts, including in Vietnam [24], China [25], and Mexico [26]. In the current study, RSES demonstrates high internal consistency (Cronbach’s α = 0.89).

Life satisfaction was evaluated using the Satisfaction with Life Scale (SWLS), developed by Life satisfaction was evaluated using the Satisfaction with Life Scale (SWLS), developed by [27]. The scale includes five statements and uses a seven-point Likert format, with response anchors ranging from 1 (Strongly Disagree) to 7 (Strongly Agree). Higher scores on the scale reflect higher satisfaction with life. In this study, the SWLS demonstrated high internal consistency, with a Cronbach’s alpha of 0.91, indicating excellent reliability. The SWLS has been validated in various sociocultural settings and among adolescents, demonstrating favorable psychometric indicators [28]. Reported Cronbach’s alpha values include those from Pakistan (α = 0.90 [29]); Chile (α = 0.81 [30]); Mexico (α = 0.74 [31]); and the Arabic version (α = 0.79 [32]).

To assess the potential for common method variance (CMV) arising from the cross-sectional, self-reported design, Harman’s single-factor test was applied by entering all items into an exploratory factor analysis without rotation. The results indicated that the first factor accounted for 39% of the variance, which is below the 50% threshold, suggesting that common method bias is not a significant concern.

### 2.4. Data Collection

Data were collected via an online questionnaire distributed primarily through WhatsApp between January and March 2025. The survey was hosted on a secure platform designed to protect confidentiality and ensure anonymity. Prior to starting the questionnaire, respondents were provided with detailed study information, confidentiality assurances, and informed consent documents emphasizing voluntary participation and the right to withdraw.

For minors, the online form required parental or guardian electronic consent prior to adolescent assent and survey completion, ensuring compliance with ethical standards for research involving children and adolescents.

### 2.5. Statistical Analysis

SPSS version 26 was used for data analysis. The chi-squared test (χ^2^) and Fisher’s exact test were applied to investigate associations between variables. Quantitative variables were expressed as mean and standard deviation (mean ± SD) and tested for normality using the One-Sample Kolmogorov–Smirnov Test, which indicated non-parametric distribution. Consequently, the Kruskal–Wallis and Mann–Whitney U tests were employed to examine relationships between non-parametric variables. Spearman’s test was used for correlation analysis.

Multivariate logistic regression analysis was performed to assess factors associated with poor mental well-being. The odds ratio (OR) was at a 95% confidence interval (CI). The OR describes the probability. A *p*-value less than 0.05 was considered to indicate statistical significance.

### 2.6. Ethical Considerations

Ethical approval was granted by the Institutional Review Board of the Faculty of Nursing at King Abdulaziz University (NREC Serial No.: Ref. No. 1B.67). All respondents were informed about the voluntary nature of participation and their right to withdraw at any time without penalty. For participants under 18, parental or guardian electronic consent was obtained, followed by age-appropriate adolescent assent, after clear explanations about the study’s nature and procedures were provided.

All data were anonymized to protect confidentiality. The study adhered to ethical guidelines for research with minors and ensured participant safety throughout data collection. Participants were provided with information to access psychological support services should distress arise. No coercion was applied during recruitment, and participants were not compensated except for reasonable reimbursement for their time.

## 3. Results

Of the 502 participants included in the study, 75.3% were aged between 16 and 18 years. Females constituted 85.9% of the sample, and the majority (81.9%) were enrolled in secondary school. Parental educational attainment was relatively high, with 41.6% of fathers and 41.0% of mothers holding university degrees. Employment status varied notably by parent gender, as 80.7% of fathers were employed compared to only 34.1% of mothers. Approximately one-third of participants reported a monthly family income ranging from 1000 to 20,000 SR. Most participants (88.2%) lived with both parents, and a substantial majority (93.2%) resided in urban areas. Notably, 11.4% of respondents indicated previous contact with a psychologist (see Table 1).

**Figure 1:**
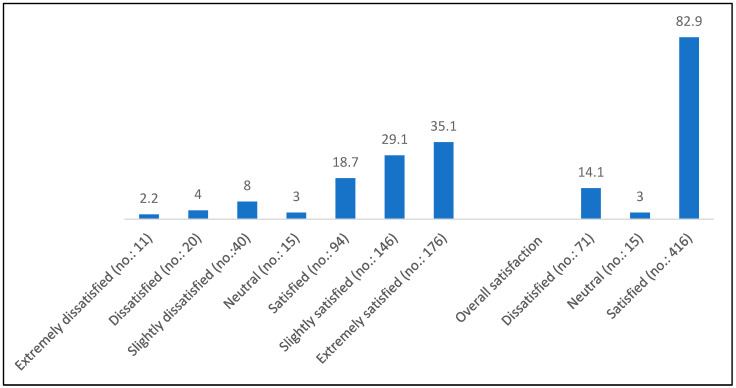
Percentage distribution of the level of life satisfaction among studied participants based on the Satisfaction with Life Scale total score classification (No.: 502).

The means and standard deviations of the administered scales are presented in Table 2. The mean total score on the SWLS was 26.84 ± 6.78, while the Rosenberg Self-Esteem (RSE) scale yielded a mean total score of 20.36 ± 5.69. According to established cut-off criteria for Satisfaction with Life Scale, 82.9% of participants were classified as satisfied with their lives, whereas 14.5% were classified as dissatisfied (Figure 1). Regarding self-esteem, 12.2% of participants (*n* = 61) fell within the low self-esteem category based on RSE classification.

Table 3 demonstrates that a significantly higher prevalence of life satisfaction was observed among secondary school students, participants from families with a monthly income of ≥10,000 SR, those living with both parents, and individuals who had never sought consultation with a psychologist (*p* < 0.05).

Table 4 outlines the association between Rosenberg Self-Esteem (RSE) classification (normal/high vs. low) and various demographic factors and prior psychological consultation among the 502 participants. No statistically significant differences in self-esteem levels were observed across age groups (χ^2^ = 0.43, *p* = 0.512), gender (χ^2^ = 0.87, *p* = 0.352), child educational level (χ^2^ = 1.41, *p* = 0.533), parental educational attainment (father: χ^2^ = 7.64, *p* = 0.174; mother: χ^2^ = 1.24, *p* = 0.945), parental employment status (father: χ^2^ = 0.18, *p* = 0.675; mother: χ^2^ = 0.86, *p* = 0.353), monthly family income (χ^2^ = 0.19, *p* = 0.979), or living arrangements (χ^2^ = 1.12, *p* = 0.795).

A marginally non-significant trend was observed with residence type, where a higher proportion of low self-esteem participants resided in rural areas compared to urban (13.1% vs. 5.9%, Fisher’s exact test *p* = 0.052). Notably, participants with low self-esteem were significantly more likely to have previously contacted a psychologist compared to those with normal/high self-esteem (19.7% vs. 10.2%, χ^2^ = 4.77, *p* = 0.029).

Moreover, a highly significant association was found between self-esteem and overall life satisfaction (χ^2^ = 46.78, *p* < 0.001), with 42.6% of participants with low self-esteem reporting dissatisfaction with life compared to only 10.2% of those with normal/high self-esteem. Conversely, 86.8% of participants with normal/high self-esteem were satisfied with their lives, compared to 54.1% in the low self-esteem group.

**Figure 2:**
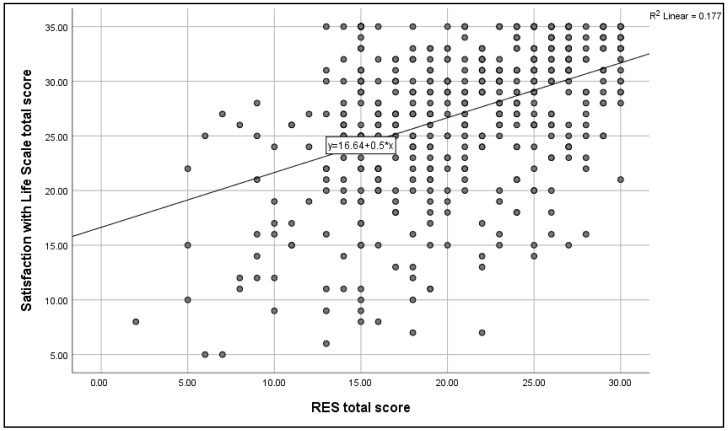
Spearman’s correlation analysis between the Satisfaction with Life Scale scores and the RES scores. N.B.: (*r* = 0.37, *p*-value < 0.001).

Figure 2 illustrates a highly significant positive correlation between Satisfaction with Life Scale scores and Rosenberg Self-Esteem (RSE) scores (r = 0.37, *p* < 0.001).

Table 5 presents the association between Rosenberg Self-Esteem (RSE) classification (normal/high vs. low) and detailed levels of life satisfaction among the 502 participants. A highly significant difference was observed across all levels of life satisfaction (χ^2^ = 14.81, *p* < 0.001). Specifically, participants with low self-esteem were disproportionately represented among those reporting extreme dissatisfaction (9.8% vs. 1.1%) and dissatisfaction (14.8% vs. 2.5%) compared to their normal/high self-esteem counterparts. Conversely, those with normal/high self-esteem constituted the majority of participants who were extremely satisfied (39.0% vs. 6.6%).

When examining overall life satisfaction categories, the prevalence of dissatisfaction was significantly greater among participants with low self-esteem (42.6% vs. 10.2%; χ^2^ = 16.77, *p* < 0.001), while satisfaction was more commonly reported in the normal/high self-esteem group (86.8% vs. 54.1%).

Table 6 presents the association between mean Rosenberg Self-Esteem total scores and detailed levels of life satisfaction among the 502 participants. Analysis of mean RSE total scores across different levels of life satisfaction did not reveal statistically significant differences (F = 0.327, *p* = 0.930). Mean RSE scores were generally consistent across varying satisfaction groups, ranging from 20.52 ± 5.68 in the extremely satisfied group to 23.0 ± 2.92 in the neutral group. Similarly, no significant difference in mean RSE scores was observed across the overall satisfaction categories (F = 0.280, *p* = 0.868).

Table 7 presents the multivariate logistic regression exploring predictors of low self-esteem among the 502 study participants. After controlling for potential confounding variables, overall life dissatisfaction emerged as the sole statistically significant independent predictor of low self-esteem, with dissatisfied participants exhibiting over seven-fold increased odds of low self-esteem compared to those who were neutral or satisfied (OR = 7.20; 95% CI: 3.75–13.83; *p* < 0.001).

Other variables were statistically insignificant—including age (16–18 years vs. 13–15 years; OR = 1.93; 95% CI: 0.68–5.40; *p* = 0.21), gender (male vs. female; OR = 1.32; 95% CI: 0.59–2.95; *p* = 0.48), child educational level (primary/middle vs. secondary; OR = 2.30; 95% CI: 0.70–7.55; *p* = 0.16), parental education, parental employment status, monthly family income, residence (rural vs. urban; OR = 2.26; 95% CI: 0.88–5.84; *p* = 0.09), and history of psychological consultation (OR = 1.68; 95% CI: 0.76–3.71; *p* = 0.19).

## 4. Discussion

In this study, the relationship between adolescents’ perceived self-esteem and life satisfaction was examined. The potential moderating effects of demographic factors on this association were also assessed. Several notable findings emerged from the analysis.

First, the study identified a statistically significant positive association between self-esteem and life satisfaction, highlighting the importance of self-concept in shaping subjective well-being during adolescence. This result is in agreement with the results of the previous study, which noted that adolescents with elevated self-esteem typically reported higher life satisfaction, supporting a widely established relationship [13,14]. This result also supports the work of studies conducted in Vietnam [24], Pakistan [29], and multi-country adolescent samples [28], which further confirms the universal relevance of self-esteem as a psychological resource.

Multivariate logistic regression revealed that adolescents reporting dissatisfaction with life exhibited lower self-esteem, supporting evidence from previous studies that identify a bidirectional relationship between these variables [14,24]. This finding aligns with self-determination theory [33], which posits that self-esteem reflects an individual’s sense of competence and autonomy—both critical for psychological well-being and life satisfaction. Importantly, this study extends the empirical understanding of this relationship to a Saudi adolescent population, which is underrepresented in psychological research. Cultural factors unique to this group may influence the dynamics of self-worth and life satisfaction, highlighting the need for culturally contextualized interpretations and interventions.

Second, adolescents from families with a monthly income exceeding 10,000 SR reported higher life satisfaction compared to those from lower-income households. This likely reflects the economic advantage that facilitates access to supportive resources, educational opportunities, and psychosocial stability, all of which contribute to more positive life evaluations. These findings align with prior studies demonstrating that individuals from higher-income households experience better psychological well-being, primarily due to greater access to social and emotional resources [34,35,36]. Additionally, income has been identified as a key determinant of life quality, often mediated through the availability of social capital [37]. In the Saudi context, where family structures and economic conditions interact in distinctive ways, this relationship is especially pertinent. Financial stress has been shown to negatively affect familial mental health dynamics, which may subsequently influence adolescents’ subjective assessments of their lives [38]. Furthermore, income disparities have been linked to differences in adolescents’ self-concept, a significant component of life satisfaction [39]. However, some research indicates that adults in lower-income quintiles exhibit higher odds of life dissatisfaction, while household income may not significantly relate to adolescents’ happiness or depression [39]. These observations suggest that, although income correlates with life satisfaction in adults, additional factors such as parenting style and healthcare quality play important roles in overall well-being, underscoring the need for further research to explore these complex interactions.

Third, educational level was found to significantly influence adolescents’ perceived life satisfaction. This association suggests that developmental transitions characteristic of this stage—including increased cognitive autonomy; the formation of stable peer relationships; and a strengthened personal identity—may play a pivotal role in shaping self-esteem and overall life satisfaction. The findings align with previous research emphasizing the importance of educational transitions in adolescent identity development. For example, Negru-Subtirica (2024) noted that during secondary school, adolescents experience increased cognitive autonomy and form consistent social bonds, which contribute to their identity formation [40]. Transitions from primary to secondary and tertiary education represent critical periods for educational identity development. Similarly, psychosocial shifts during adolescence have been theorized to enhance life satisfaction [5,11,33]. Although the relationship between education and well-being has been examined globally, its specific manifestations within Middle Eastern adolescent populations remain insufficiently documented, underscoring the contextual significance of the present findings.

Additionally, the results indicated that adolescents living with both parents reported significantly higher life satisfaction, suggesting that intact family structures contribute to greater emotional security and psychological resilience. Previous research has shown that cohabitation with both parents promotes consistent support, increased parental involvement, and reduced exposure to familial conflict, all of which are linked to improved mental health outcomes [6,41]. Within the Saudi cultural context, where family unity holds considerable socio-religious importance, this relationship may be especially pronounced. However, these results should be interpreted with caution, as cultural factors were not directly assessed in this study, despite their likely influence on family dynamics and adolescents’ life satisfaction. Overall, the findings underscore the lasting significance of familial and educational environments in shaping adolescent well-being.

In the Saudi cultural context, living with both parents represents more than a family structure variable; it reflects deeply rooted religious and cultural norms that emphasize family unity, cohesion, and collective support [4,38,42]. This familial arrangement provides not only structural stability but also vital social and emotional support, which significantly influences adolescents’ life satisfaction and self-esteem [4,38,42]. The societal emphasis on intact family units underscores values that regard family togetherness as fundamental to psychological resilience and well-being. Compared to settings where family dynamics may be more variable or less culturally reinforced, the positive effects of living with both parents in Saudi Arabia may be particularly pronounced. While these findings are consistent with global research on the advantages of intact families, future research should explore how family quality and specific sociocultural expectations further shape adolescent life satisfaction across diverse populations.

The study found that prior contact with a psychologist was associated with both lower self-esteem and lower life satisfaction. This finding suggests that psychological interventions may not immediately result in improvements in adolescents’ well-being, especially when underlying emotional or behavioral difficulties persist. These results are consistent with Cavioni et al. (2021), who noted that initial therapeutic support often requires extended engagement before significant enhancements in life satisfaction are observed [11]. Additionally, the complexity of adolescent mental health concerns, including anxiety, depression, or trauma, may not be fully addressed through short-term interventions [5].

Cultural attitudes toward mental health and psychological services significantly shape adolescent experiences in Saudi Arabia. In the present study, prior contact with a psychologist, measured as a behavioral indicator, likely reflects deeper cultural dynamics, including stigma, social perceptions, and structural barriers that impede sustained engagement with psychological care. Empirical research within Saudi and Gulf contexts has documented that mental health stigma is often intertwined with concerns about family honor, gender roles, and religious beliefs, which collectively discourage help-seeking behaviors, particularly among youth [4,17,20]. These culturally embedded factors may partially explain the observed association between prior psychological consultation and lower well-being in this sample, although causality cannot be inferred due to the cross-sectional design. It is also plausible that prior contact with a psychologist may indicate preexisting severity of psychological distress rather than represent a direct cause of reduced well-being. Addressing these challenges requires culturally adapted, long-term support strategies. For example, research suggests that interventions tailored to the cultural context—such as integrating family engagement into therapy; enhancing community mental health literacy programs; and training psychologists in culturally competent care—can help reduce stigma and foster trust [1,5,6]. Collaboration with religious leaders, incorporation of culturally relevant practices, and targeting social perceptions have also been found effective in increasing access and acceptability of mental health services in Saudi Arabia.

Future research should empirically examine the role of sociocultural moderators—including values, norms, and religious beliefs—in mental health service utilization and intervention outcomes among Saudi adolescents. The absence of direct evaluation of cultural constructs within the present investigation circumscribes the capacity to ascertain their nuanced moderating influences, thereby accentuating the imperative of integrating psychometrically robust cultural indices in subsequent inquiries to more incisively delineate their role in modulating help-seeking behaviors, engagement, and psychological well-being. Previous studies have emphasized the importance of understanding these cultural nuances, as adolescents in the region often encounter unique barriers to fully engaging with psychological services, such as social stigma and societal expectations [21,22]. These factors may help explain why prior psychological therapy is associated with lower well-being in this sample, as adolescents may not receive continuous, effective support or may encounter additional psychological and social obstacles. The need remains for long-term, culturally sensitive interventions to enhance psychological outcomes in adolescent populations, particularly in Middle Eastern contexts, where family and societal pressures profoundly influence adolescent development [16].

Finally, the results indicated that dissatisfaction with life was the only factor demonstrating a statistically significant independent association with low self-esteem. Adolescents reporting life dissatisfaction were over seven times more likely to exhibit low self-esteem. Consistent with Usán Supervía, et al. (2023), self-esteem has been identified as a significant mediator between resilience and life satisfaction, as well as between self-efficacy and life satisfaction [2]. Additionally, self-esteem mediates the relationship between empathy and life satisfaction. Further research supports the role of self-esteem as a protective buffer in the relationship between adverse life experiences and mental health outcomes during adolescence [14,15]. Understanding the significance of self-esteem in these contexts can guide the development of interventions aimed at fostering positive youth development and mental well-being. These findings also align with self-determination theory [33], which posits that deficits in competence and autonomy undermine psychological resilience, potentially perpetuating negative cycles affecting self-esteem and overall well-being.

While prior research has established that low self-esteem predicts lower well-being [11], this study provides evidence for the reverse relationship, identifying dissatisfaction with life as a significant risk factor for internalized low self-worth. This underscores the importance of early identification of life dissatisfaction as a potential intervention point to prevent further psychological decline. Future research should investigate the long-term effects of such interventions and examine how sociocultural factors, including peer relationships and family dynamics, may moderate the relationship between life dissatisfaction and self-esteem.

### 4.1. Strengths and Limitations

This study offers several strengths that enhance the credibility of its findings. With a large, diverse sample of 502 adolescents across Saudi Arabia, the study achieved strong statistical power and socioeconomic representation. The use of validated psychometric instruments added methodological rigor and facilitated cross-cultural comparability. The study also contributes novel data from a region that remains underrepresented in global adolescent mental health research.

However, certain limitations must be acknowledged. The cross-sectional design precludes any conclusions about causality, limiting interpretations of directionality between self-esteem and life satisfaction. Additionally, the use of self-reported online surveys introduces potential response and social desirability biases, especially on sensitive mental health indicators. The convenience sampling method, while practical for reaching adolescents across platforms like WhatsApp and Telegram, may have introduced selection bias, possibly overrepresenting more tech-literate or urban respondents. Direct cultural constructs, such as values, norms, and belief systems, were not measured in this study; therefore, cultural interpretations are based on theoretical and contextual insights drawn from prior literature. Future research should incorporate validated cultural measures to more accurately assess how culture influences self-esteem and life satisfaction among Saudi adolescents.

Finally, mental health history was captured only as prior contact with a psychologist, which may underestimate undiagnosed or unreported conditions. These limitations highlight the need for cautious generalization and underscore the importance of future studies using longitudinal and mixed-method designs.

### 4.2. Implications

These findings support the view that self-esteem operates as a fundamental psychological driver of adolescent well-being. That adolescents who viewed their lives unfavorably were also more likely to exhibit diminished self-worth suggests a deeper interplay between how young adolescents felt about their lives and how they perceived themselves. Rather than treating self-esteem solely as a precursor to well-being, this dynamic points toward a more reciprocal relationship—one in which dissatisfaction may erode self-concept, and low self-esteem in turn may color how life circumstances are interpreted. Such patterns resonate with transactional models of development, which emphasize the ongoing interaction between internal psychological states and external experiences.

Interestingly, some expected demographic predictors—like gender and parental education—did not appear to influence self-esteem or life satisfaction. This may indicate that in certain cultural contexts, such as Saudi Arabia, other social factors (e.g., family cohesion or perceived stability) play a more dominant role in shaping these outcomes. Theoretical frameworks concerned with adolescent development, therefore, should be expanded to consider how culturally situated experiences mediate these well-established psychological processes.

## 5. Conclusions

This study reinforces that self-esteem is a critical determinant of life satisfaction among adolescents, with dissatisfaction emerging as a particularly strong risk factor for reduced self-worth. These findings, drawn from a large and demographically diverse Saudi sample, contribute unique regional evidence to a predominantly Western literature. Socioeconomic and family structure factors also influenced life satisfaction, highlighting the importance of both individual and contextual factors in adolescent mental health. The strength of the association between dissatisfaction and low self-esteem suggests that routine screening for subjective well-being in schools may offer an effective early detection strategy.

Although demographic and socioeconomic variables in this study provide insight into cultural influences on adolescent self-esteem and life satisfaction, direct measures of cultural constructs—such as values; norms; and identity—were not included. Consequently, assertions about cultural factors sustaining these relationships are based on theoretical and contextual knowledge rather than direct empirical evidence from the data. Future research incorporating validated cultural assessments would enhance understanding of how these sociocultural dimensions intricately impact adolescent psychological outcomes in Saudi and similar Middle Eastern populations.

## Figures and Tables

**Table 1:** Distribution of studied participants according to their demographic characteristics and contacting a psychologist in the past (No.: 502).

Variable	No. (%)
Age (years)	
13–15	124 (24.7)
16–18	378 (75.3)
Gender	
Female	431 (85.9)
Male	71 (14.1)
Child educational level	
Primary	10 (2)
Middle	81 (16.1)
Secondary	411 (81.9)
Father’s educational level	
Illiterate	7 (1.4)
Primary	18 (3.6)
Middle	55 (11)
Secondary	161 (32.1)
University	2019 (41.6)
Postgraduate	52 (10.4)
Mother’s educational level	
Illiterate	17 (3.4)
Primary	31 (6.2)
Middle	47 (9.4)
Secondary	167 (33.3)
University	206 (41)
Postgraduate	34 (6.8)
Father’s employment status	
Unemployed	97 (19.3)
Employed	405 (80.7)
Mother’s employment status	
Unemployed	331 (65.9)
Employed	171 (34.1)
Monthly family income	
<5000 SR	60 (12)
5000–<10,000 SR	129 (25.7)
10,000–20,000 SR	155 (30.9)
>20,000 SR	158 (31.5)
Living status	
Living with other relatives	4 (0.8)
Living with only the father	14 (2.8)
Living with only the mother	41 (8.2)
Living with both parents	443 (88.2)
Residence	
Rural	34 (6.8)
Urban	468 (93.2)
Have you ever contacted a psychologist?	
No	445 (88.6)
Yes	57 (11.4)

**Table 2:** Mean and SD of the used scales.

Scale	Mean ± SD
Satisfaction with Life Scale total score	26.84 ± 6.78
Rosenberg’s Self-Esteem scale (RSE) total score	20.36 ± 5.69

**Table 3:** Relationship between life satisfaction level and participants’ demographic characteristics and contacting a psychologist in the past (No.: 502).

Variable	Overall Life Satisfaction			χ2	*p*-Value
	Dissatisfied No. (%)	Neutral No. (%)	Satisfied No. (%)		
Age (years)					
13–15	15 (21.1)	1 (6.7)	108 (26)	3.46	0.177
16–18	56 (78.9)	14 (93.3)	308 (74)		
Gender					
Female	63 (88.7)	13 (86.7)	355 (85.3)	0.58	0.747
Male	8 (11.3)	2 (13.3)	61 (14.7)		
Child educational level					
Primary	0 (0.0)	0 (0.0)	10 (2.8)	10.85	0.028
Middle	4 (5.6)	1 (6.7)	76 (18.3)		
Secondary	67 (94.4)	14 (93.3)	330 (79.3)		
Father’s educational level					
Illiterate	3 (4.2)	0 (0.0)	4 (1)	14.62	0.146
Primary	4 (5.6)	1 (6.7)	13 (3.1)		
Middle	11 (15.5)	2 (13.3)	42 (10.1)		
Secondary	23 (32.4)	7 (46.7)	131 (31.5)		
University	21 (29.6)	3 (20)	185 (44.5)		
Postgraduate	9 (12.7)	2 (13.3)	41 (9.9)		
Mother’s educational level					
Illiterate	2 (2.8)	2 (13.3)	13 (3.1)	16.18	0.094
Primary	6 (8.5)	1 (6.7)	24 (5.8)		
Middle	8 (11.3)	3 (20)	36 (8.7)		
Secondary	24 (33.8)	8 (53.3)	135 (32.5)		
University	25 (35.2)	1 (6.7)	180 (43.3)		
Postgraduate	6 (8.5)	0 (0.0)	28 (6.7)		
Father’s employment status					
Unemployed	20 (28.2)	3 (20)	74 (17.8)	4.19	0.123
Employed	51 (71.8)	12 (80)	342 (82.2)		
Mother’s employment status					
Unemployed	53 (74.6)	12 (80)	266 (63.9)	4.45	0.106
Employed	13 (25.4)	3 (20)	150 (36.1)		
Monthly family income					
<5000 SR	16 (22.5)	4 (26.7)	40 (9.6)	16.18	0.013
5000–<10,000 SR	22 (31)	3 (20)	104 (25)		
10,000–20,000 SR	15 (21.1)	4 (26.7)	136 (32.7)		
>20,000 SR	18 (25.4)	4 (26.7)	136 (32.7)		
Living status					
Living with other relatives	0 (0.0)	1 (6.7)	3 (0.7)	15.74	0.015
Living with only the father	0 (0.0)	1 (6.7)	13 (7.1)		
Living with only the mother	11 (15.5)	1 (6.7)	29 (7)		
Living with both parents	60 (84.5)	12 (80)	371 (89.2)		
Residence					
Rural	9 (12.7)	0 (0.0)	25 (6)	5.29	0.067
Urban	62 (87.3)	15 (100)	391 (94)		
Have you ever contacted a psychologist?					
No	56 (78.9)	13 (86.7)	376 (90.4)	8.04	0.018
Yes	15 (21.1)	2 (13.3)	40 (9.6)		

**Table 4:** Relationship between the self-esteem levels based on the RES scale classification and participants’ demographic characteristics and contacting a psychologist in the past (No.: 502).

Variable	Self-Esteem		χ^2^	*p*-Value
	Normal and High	Low		
Age (years)				
13–15	111 (25.2)	13 (21.3)	0.43	0.512
16–18	330 (74.8)	48 (78.7)		
Gender				
Female	381 (86.4)	50 (82.0)	0.87	0.352
Male	60 (13.6)	11 (18.0)		
Child educational level				
Primary	10 (2.3)	0 (0)	1.41	0.533
Middle	71 (16.1)	10 (16.4)		
Secondary	360 (81.6)	51 (83.6)		
Father’s educational level				
Illiterate	5 (1.1)	2 (3.3)	7.64	0.174
Primary	17 (3.9)	1 (1.6)		
Middle	47 (10.7)	8 (13.1)		
Secondary	144 (32.7)	17 (27.9)		
University	187 (42.4)	22 (36.1)		
Postgraduate	41 (9.3)	11 (18.0)		
Mother’s educational level				
Illiterate	16 (3.6)	1 (1.6)	1.24	0.945
Primary	26 (5.9)	5 (8.2)		
Middle	42 (9.5)	5 (8.2)		
Secondary	147 (33.3)	20 (32.8)		
University	180 (40.8)	26 (42.6)		
Postgraduate	30 (6.8)	4 (6.6)		
Father’s employment status				
Unemployed	84 (19.0)	13 (21.3)	0.18	0.675
Employed	357 (81.0)	48 (78.7)		
Mother’s employment status				
Unemployed	294 (66.7)	37 (60.7)	0.86	0.353
Employed	147 (33.3)	24 (39.3)		
Monthly family income				
<5000 SR	53 (12.0)	7 (11.5)	0.19	0.979
5000–<10,000 SR	113 (25.6)	16 (26.2)		
10,000–20,000 SR	135 (30.6)	20 (32.8)		
>20,000 SR	140 (31.7)	18 (29.5)		
Living status				
Living with other relatives	4 (0.9)	0 (.00)	1.12	0.795
Living with only the father	13 (2.9)	1 (1.6)		
Living with only the mother	35 (7.9)	6 (9.8)		
Living with both parents	389 (88.2)	54 (88.5)		
Residence				
Rural	26 (5.9)	8 (13.1)	* 4.42	0.052
Urban	415 (94.1)	53 (86.9)		
Have you ever contacted a psychologist?				
No	396 (89.8)	49 (80.3)	4.77	0.029
Yes	45 (10.2)	12 (19.7)		
Overall Satisfaction				
Dissatisfied	45 (10.2)	26 (42.6)	46.78	<0.001
Neutral	13 (2.9)	2 (3.3)		
Satisfied	383 (86.8)	33 (54.1)		

N.B.: * = Fisher’s exact test.

**Table 5:** Relationship between the self-esteem levels based on the RES scale classification and levels of life satisfaction (No.: 502).

Variable	Self-Esteem		χ^2^	*p*-Value
	Normal and High	Low		
All levels of life satisfaction				
Extremely dissatisfied	5 (1.1)	6 (9.8)	14.81	<0.001
Dissatisfied	11 (2.5)	9 (14.8)		
Slightly dissatisfied	29 (6.6)	11 (18)		
Neutral	13 (2.9)	2 (3.3)		
Satisfied	80 (18.1)	14 (23)		
Slightly satisfied	131 (29.7)	15 (24.6)		
Extremely satisfied	172 (39)	4 (6.6)		
Overall satisfaction				
Dissatisfied	45 (10.2)	26 (42.6)	16.77	<0.001
Neutral	13 (2.9)	2 (3.3)		
Satisfied	383 (86.8)	33 (54.1)		

**Table 6:** Relationship between mean Rosenberg Self-Esteem total scores and levels of life satisfaction (No.: 502).

Variable	RSE Total Score (Mean ± SD)		
All levels of life satisfaction			
Extremely dissatisfied	22.72 ± 5.62	6.93	0.327
Dissatisfied	21.8 ± 3.92		
Slightly dissatisfied	22.3 ± 3.31		
Neutral	23 ± 2.92		
Satisfied	22.75 ± 3.43		
Slightly satisfied	22.65 ± 3.66		
Extremely satisfied	20.52 ± 5.68		
Overall satisfaction			
Dissatisfied	22.22 ± 3.86	0.28	0.868
Neutral	23 ± 2.92		
Satisfied	21.77 ± 4.7		

**Table 7:** Multivariate logistic regression analysis of risk factors of low self-esteem among studied participants.

Variable	The Multivariate analysis					
	B	OR	SE	*p*-Value	95% CI	
					Lower	Upper
Age 16–18 13–15 (Reference)	0.66	1.93	0.53	0.21	0.68	5.4
Gender Male Female (Reference)	0.28	1.32	0.4	0.48	0.59	2.95
Child educational level Primary and middle Secondary (Reference)	0.83	2.3	0.60	0.16	0.7	7.55
Father’s educational level University and post-graduate secondary or less education (Reference)	0.25	1.28	0.33	0.44	0.67	2.45
Mother’s educational level Secondary or less education University and post-graduate (Reference)	0.13	1.15	0.34	0.68	0.58	2.26
Father’s employment status Unemployed Employed (Reference)	0.03	1.03	0.0	0.94	0.47	2.25
Mother’s employment status Employed Unemployed (Reference)	0.42	1.53	0.34	0.21	0.78	2.98
Monthly family income 10,000–>20,000 SR <5000–<10,000 SR (Reference)	0.06	1.07	0.37	0.85	0.51	2.21
Living status Living with other relatives Living off one or both parents (Reference)	−18.67	0.00	3.926	0.99	0.00	0.00
Residence Rural Urban (Reference)	0.81	2.26	0.48	0.09	0.88	5.84
Have you ever contacted a psychologist? Yes No (Reference)	0.52	1.68	0.4	0.19	0.76	3.71
Overall Satisfaction Dissatisfied Neutral and satisfied (Reference)	1.97	7.2	0.33	<0.001	3.75	13.83

OR: Odds ratio—SE: Standard error—CI: Confidence interval.

## Data Availability

The datasets generated and analyzed during the current study are not publicly available for data security but are available from the corresponding author on reasonable request.
